# Chimeric non-antigen receptors in T cell-based cancer therapy

**DOI:** 10.1136/jitc-2021-002628

**Published:** 2021-08-03

**Authors:** Jitao Guo, Andrew Kent, Eduardo Davila

**Affiliations:** 1 Division of Medical Oncology, Department of Medicine, University of Colorado - Anschutz Medical Campus, Aurora, Colorado, USA; 2 Human Immunology and Immunotherapy Initiative, University of Colorado, Anschutz Medical Campus, Aurora, Colorado, USA; 3 University of Colorado Comprehensive Cancer Center, Aurora, Colorado, USA; 4 Department of Medicine, University of Colorado, Anschutz Medical Campus, Aurora, Colorado, USA

**Keywords:** cell engineering, costimulatory and inhibitory T-cell receptors, cytotoxicity, immunologic, immunotherapy, lymphocyte activation

## Abstract

Adoptively transferred T cell-based cancer therapies have shown incredible promise in treatment of various cancers. So far therapeutic strategies using T cells have focused on manipulation of the antigen-recognition machinery itself, such as through selective expression of tumor-antigen specific T cell receptors or engineered antigen-recognition chimeric antigen receptors (CARs). While several CARs have been approved for treatment of hematopoietic malignancies, this kind of therapy has been less successful in the treatment of solid tumors, in part due to lack of suitable tumor-specific targets, the immunosuppressive tumor microenvironment, and the inability of adoptively transferred cells to maintain their therapeutic potentials. It is critical for therapeutic T cells to overcome immunosuppressive environmental triggers, mediating balanced antitumor immunity without causing unwanted inflammation or autoimmunity. To address these hurdles, chimeric receptors with distinct signaling properties are being engineered to function as allies of tumor antigen-specific receptors, modulating unique aspects of T cell function without directly binding to antigen themselves. In this review, we focus on the design and function of these chimeric non-antigen receptors, which fall into three broad categories: ‘inhibitory-to-stimulatory’ switch receptors that bind natural ligands, enhanced stimulatory receptors that interact with natural ligands, and synthetic receptor-ligand pairs. Our intent is to offer detailed descriptions that will help readers to understand the structure and function of these receptors, as well as inspire development of additional novel synthetic receptors to improve T cell-based cancer therapy.

## Introduction

**Figure 1:**
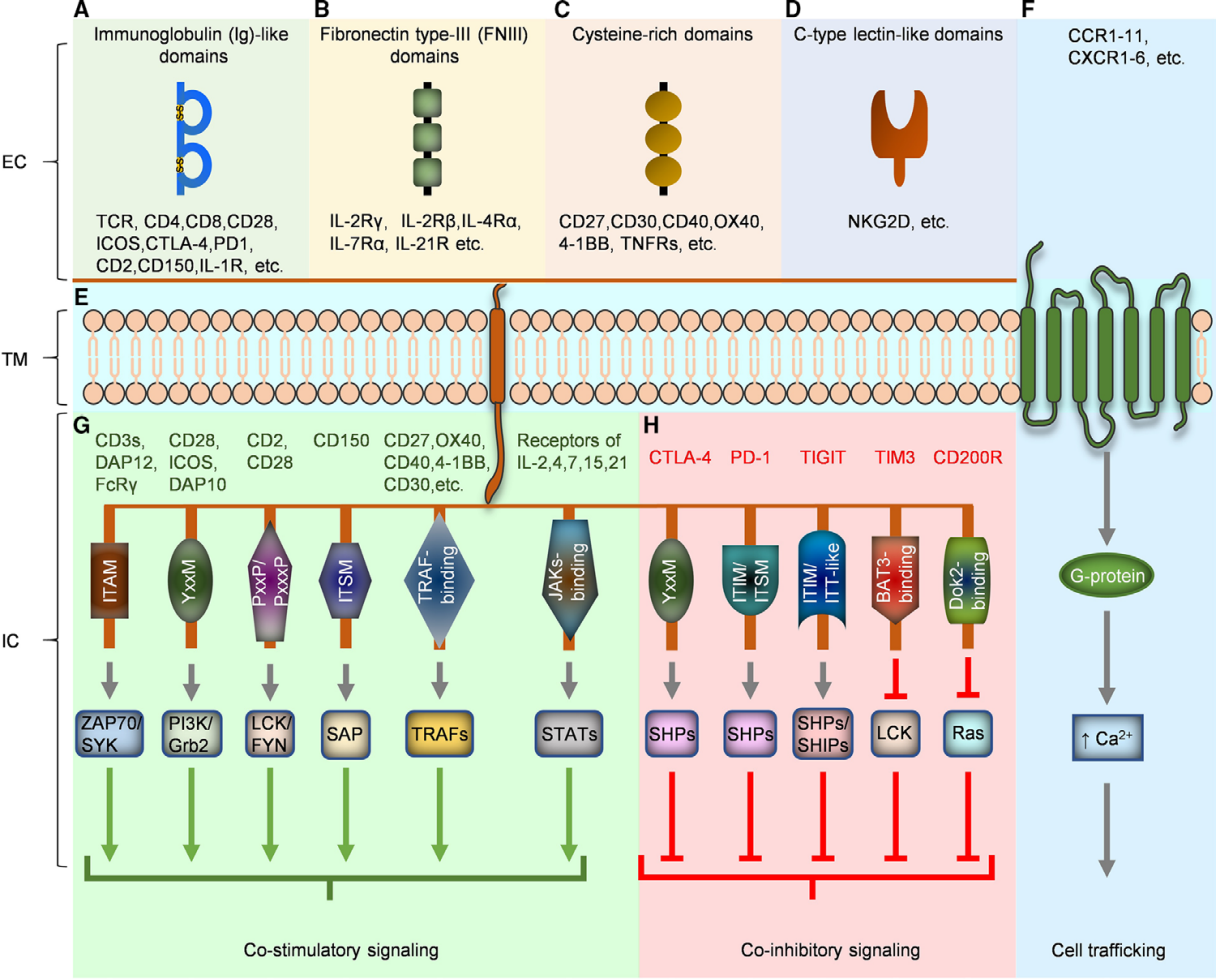
Functional subunits of natural immune receptor related to regulation of antitumor TCRs. Intracellular (IC) (EC), transmembrane (TM), IC, Green arrow (activation), red line (inhibition). Colored rectangles represent subsections of subunits: Ig-like domains (A), FNIII domains (B), Cysteine-rich domains (C), C-type lectin-like domains (D), TM domains (E), 7-TM immune receptors (F), IC domains/motifs inducing costimulatory signaling (G), and IC domains/motifs inducing co-inhibitory signaling (H). SHP, SH2-containing phosphatases; TCR, T cell receptor; TRAFs, TNR receptor-associated factors.

**Figure 2:**
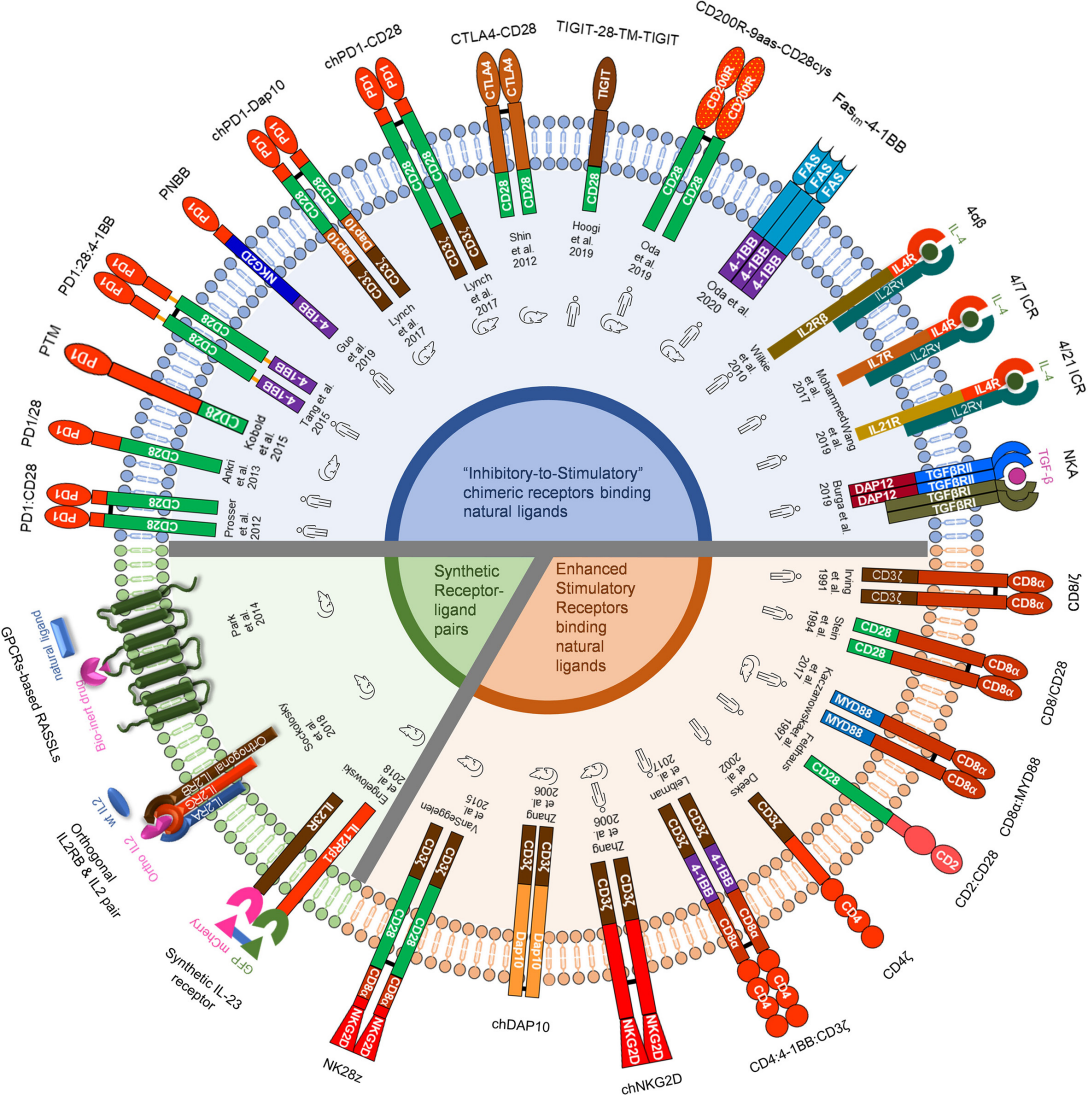
Schematic diagrams of chimeric non-antigen receptors for T cell-engineering in cancer therapy. Receptors are classified into three group as ‘inhibitory to stimulatory’ switch receptors binding natural ligands (blue), enhanced stimulatory receptors binding natural ligands (brown), and synthetic receptor-ligand pair (green). Disulfide bond (black solid line), linkers (yellow solid line), and dimeric fusion GFP/mCherry (blue solid line) are indicated. Whether the synthetic receptors were of human or mouse in origin is denoted with icons. IL2, interleukin 2.

Diverse types of immunotherapies are under development to use a patient’s own immune system to fight cancer. Engineering of T cells expressing tumor-reactive T cell receptors (TCRs) or chimeric antigen receptors (CARs) has achieved great results, with U.S. Food and Drug Administration(FDA)-approval of three CAR T therapies for B cell malignancies since 2017.^1 2^ The preeminent aim of T cell bioengineering in cancer immunotherapy is to direct the desired proinflammatory and cytotoxic effects of T cells toward tumor cells, while at the same time preventing unwanted off-target effects or misdirected inflammation. Precise regulation of all parameters, including T cell trafficking, survival, proliferation, differentiation, and effector functions, would ideally fall under user-directed customizable control. With these goals in mind, chimeric non-antigen receptors are being designed to provide supportive cosignaling for CAR or TCR antitumor T cell responses. The domains, smaller motifs, and even key residues of natural immune receptors are the essential functional subunits of each receptor. Many domains and motifs exhibit high functional fidelity so long as their structural context is maintained, making them transplantable into chimeric proteins as functional modules. In this review, the function of various subunits from natural immune receptors related to regulation of T cell antitumor responses will be briefly introduced (figure 1). Next, the design details and functions of chimeric non-antigen receptors derived from these subunits will be discussed. According to function and ligand-type, these receptors are classified into three types in this review: (1) ‘inhibitory-to-stimulatory’ switch receptors that bind natural ligands, (2) enhanced stimulatory receptors interacting with biological ligands and (3) synthetic receptor-ligand pairs (figure 2).

### Functional subunits at the cell membrane

#### Extracellular domains

Four major extracellular domain archetypes are commonly incorporated into natural receptors on T cells. First, immunoglobulin (Ig)-like domains are widely shared among immune receptors for ligand recognition, including the TCR subunits, co-receptors (eg, CD4 and CD8), CD28 family of receptors (eg, CD28, ICOS, CTLA-4 and PD1), the CD2 family of receptors (eg, CD2 and CD150), and the interleukin 1 (IL-1) cytokine receptor (figure 1A).^3^ They contain 70–110 amino acids and have a compact sandwich-like structure formed by two sheets of antiparallel β strands linked by a stabilizing disulfide bond. Loops extending from the core structure usually define their ligand specificities. Second, fibronectin type-III (FNIII) domains are topologically similar to Ig-like domains.^4^ They are composed of 7 antiparallel β strands in two sheets stabilized by a core of hydrophobic residues, but in contrast to Ig-like domains do not incorporate a disulfide bond.^5^ FNIII domains exist in the extracellular portion of the common γ chain family of receptors (eg, IL-2, IL-7, IL-15, and IL-21)(figure 1B).^6^ The third type of highly shared ectodomain is the cysteine rich domain of the tumor necrosis factor receptor (TNFR) superfamily, which includes costimulatory receptors like CD27, CD30, CD40, OX40, and 4-1BB, as well as the TNF cytokine receptors (figure 1C).^7^ TNFRs are characterized by 1–6 cysteine rich domains, and form trimeric complexes upon ligand recognition. TNFR extracellular domains can be coupled to intracellular TNF receptor associated factors (TRAFs) to mediate various pro-inflammatory or proliferative signals (as in CD40 and TNFR2), death domains (DD’s) to mediate cell death (as in Fas or TNFR1), or can be non-membrane bound and function independently as soluble or decoy receptors.^8^ Finally, c-type lectin receptor (CLR) domains are less common than the prior three extracellular domain types in adaptive immune cells, but could be important components of chimeric receptors as will be described later in this review. One CLR family, the NKG2, is expressed on natural killer (NK) and T cells, and modulates both pro and anti-inflammatory effects in response to stress-induced molecules on self-cells including cancer cells (figure 1D).^9^

#### Transmembrane domains

Two broad transmembrane categories are present in receptors naturally expressed on T cells: single pass (bitopic), or multipass (polytopic). Bitopic transmembrane domains are very common and contain varying numbers of hydrophobic residues that allow insertion in the hydrophobic lipid membrane, while choice hydrophilic or charged residues work in conjunction with membrane proximal disulfide bonds and extracellular domain interactions to promote polymerization. An example is the TCR complex itself with six individual CD3 molecules, the two TCR molecules and either CD4 or CD8, all with individual single pass transmembrane domains (figure 1E).^10^ Polytopic receptors, such as the prominent G-protein-coupled receptor (GPCR) family, are also common in immune cells. The GPCR family of chemokine receptors which includes CXCR1-6 and CCR1-11, are defined by seven α helical passes through the membrane forming a barrel shape (figure 1F). Their extracellular loops and N-terminus determine ligand specificity, while their intracellular loops and C-terminus are responsible for signal transduction.

#### Intracellular domains

A wide variety of intracellular domains are employed in immune receptors to mediate a huge variety of activating and inhibitory functions (figure 1G, H). In some cases, the entire domain is considered activating or inhibiting such as in the TRAF and DDs used by TNFR family members as mentioned above. Alternately, specific sequences of amino acids, or motifs, perform specific functions. Two common opposing motifs are the immunoreceptor tyrosine-based activating and inhibitory motifs (ITAMs and ITIMs, respectively).^11^ ITAMs are composed of a conserved YXXL/I(X_6-8_)YXXL/I sequence (where X denotes any amino acid). The number of ITAMs in an intracellular domain or multimeric structure is key to determine signal strength and specificity. Six ITAMs form the basis for all TCR-complex signaling, and single ITAMs represent the main signaling component of other activating receptors including DNAX activation protein of 12 kDa (DAP12) and the γ subunit of the Ig Fc receptor.^12 13^ ITIMs, in contrast, are composed of the conserved (I/V/L/S)-X-Y-X-X-(L/V) sequence and lead to suppression of T cell activation and effector functions. For example, T cell immunoreceptor with Ig and ITIM domain (TIGIT) uses an ITIM and an Ig tail tyrosine -like domain to mediate inhibitory regulation by recruiting the SH2-containing phosphatases (SHIPs and SHPs).^14^

Some motifs can be employed to mediate opposing functions depending on their context. For instance, the YXXM motif is crucial to CD28 recruitment of PI3K and Grb2 needed for co-stimulation signals, while a similar YXXM motif in CTLA-4 mediates recruitment of PI3K and the tyrosine phosphatase SHP-2, but not Grb2, ultimately leading to T cell inhibition.^15^ The intervening X residues determine this modular binding partner activity, with an asparagine residue at the +2 position being essential for Grb2 interactions.^16^ Contextual residues flanking the YXXM motif also help shape the balance of activation and inhibition. The PI3K-binding YXXM motif is also a component in common γ family of cytokine receptors such as the IL-2 receptor and receptors coupled to the tripartite motif family, DNAX-activating protein 10 (DAP10), and couple myeloid differentiation primary response protein 88 (MYD88) adapter proteins.^17^ Conserved box 1 and 2 motifs in the cytokine receptors for Janus kinase (JAK) binding, and STAT docking sites add layers of subtlety to differential cytokine signaling.

Many more features of other intracellular domains have well-defined roles. For example, CD2 enhances TCR signaling by enhancing recruitment of LCK and FYN to the immune synapse through its intracellular tails comprised of five copies of PXXP or PXXXP motifs.^18^ CD200R inhibits Ras activation by recruiting tyrosine kinase Dok2 by its phosphotyrosine-binding domain recognition motif, Asn-Pro-X-Tyr (NPXY).^19^ Finally, TIM-3 does not have a known inhibitory motif; its role in T cell regulation is dependent on phosphorylation at key tyrosine residues 265 and 272, and subsequent interaction with HLA-B-associated transcript 3.^20^

Other molecules use combinations of motifs to fine tune the balance between activation and inhibition. CD150 has two immunoreceptor tyrosine-based switch motifs (ITSM), which function through SLAM-associated protein to provide activation signals for T cell proliferation and interferon-γ production (figure 1G).^21^ Alternatively, PD1 has an ITIM and an ITSM for recruiting SHP-2 which prevents CD28-mediated PI3K activation.^22 23^

Members of the TNFR, which transduce signals via TNFR-associated factors (TRAFs), have also been exploited to promote T cell activity (figure 1G). There exist six TRAFs (TRAF1-TRAF6), which contain a conserved C-terminal TRAF domain, and can serve as mediators that link receptor activation signals to intracellular signaling proteins. There is growing literature indicating that TRAFs can enhance the effects of TCRs, costimulatory TNFRs, and cytokine receptors in T cells and play an important role in regulating their function, lineage commitment, and cell survival.

Altogether, distinct functions of immune receptors are determined by their extracellular ligand specificities and signal outputs as defined by the interactome of their intracellular tails. A dissection of receptor functions into subunits, domains, and motifs provides the basic modules for synthetic receptor design.

### ‘Inhibitory-to-stimulatory’ chimeric receptors binding natural ligands

Immunosuppressive signals within TME inhibit antitumor T cell responses through inhibitory receptors on T cells. These interactions can be blocked by neutralizing antibodies or genetic disruption of relevant receptors. An alternative approach is to switch the inhibitory output to a stimulatory one using ‘inhibitory-to-stimulatory’ chimeric receptors. Such chimeric receptors further minimize the negative effects of suppressive ligands by competing with natural inhibitory receptors. Most of such receptors are composed of ectodomains of an inhibitory receptor (either coinhibitory receptor or inhibitory cytokine receptor) paired with intracellular domains from stimulatory receptors for transducing stimulatory signaling.

#### PD1-based stimulatory chimeric receptors

PD1 is a dominant inhibitory receptor responsible for T cell exhaustion in TME. Several groups have attempted to convert the PD1 signal into a stimulatory one by fusing extracellular PD1 components with stimulatory intracellular domains from other molecules, particularly CD28. Prosser *et al* generated a human chimeric receptor called PD1:CD28 which contains the PD-1 extracellular portion (AA1-155) fused to the stalk/transmembrane/cytoplasmic tail of CD28 (AA141-220).^24^ The cysteine (AA141) in the CD28 stalk region required for homodimerization was preserved. In vitro, this human PD1:CD28 was shown to respond to programmed death-ligand 1 (PD-L1)-expressing tumor cells and transduce activation signals through the chimeric receptor to augment CD8^+^ T cell proliferation, cytokine production and cytotoxic function. Using human tumor cell lines in a xenograft mouse model, Liu *et al* showed that the PD1:CD28 receptor could be used with different CARs to boost CAR T cell activity, in aspects of tumor killing, cytokine secretion, infiltration and expansion in tumor, and downregulation of PD1 and TIM3.^25^ Ankri *et al* used a larger PD1 extracellular portion (AA1-165) fused to just the transmembrane domain and cytoplasmic tail of CD28 (AA153-220), forming a human chimeric receptor they called PD1/28.^26^ PD1/28 worked efficiently to translate the PD-L1-binding signal to activation signals in T cells, indicating that CD28 AA141 cysteine-mediated dimerization is not absolutely needed for the chimeric receptor function. Importantly, PD1/28 significantly assisted T cells expressing tumor reactive TCR DMF4 to proliferate and control tumor growth in a xenograft mouse model. Schlenker *et al* compared the performance of PD1:CD28 and PD1/28 and found no significant difference between their ability to augment T cell cytokine secretion or signaling from the synthetic receptors. Interestingly, both receptors promoted the responses of low-avidity T cells resulting in enhanced tumor cell killing. Further, responses were comparable to those of high-avidity T cells and without the need to alter TCR affinity, a finding which allows the low-avidity T cells previously thought to be therapeutically inefficient to be reconsidered for T cell-based cancer therapy. In human melanoma xenograft and murine HCC models, PD1/28 receptor augmented intratumoral T cell proliferation and shifted the cytokine balance of tumor-infiltrating lymphocytes (TILs) towards one that favored antitumor activity, thus arguing for their potential applications in solid tumors.^27^ Kobold *et al* compared a panel of murine PD1 and CD28 chimeric receptor designs, and found their PD1-transmembrane construct (PTM) containing the PD1 extracellular and transmembrane domain (AA1-190) and CD28 intracellular domain (AA178-218) was superior to murine equivalents of the PD1:CD28 and PD1/28 constructs, which contain the CD28 transmembrane domain.^28^ It remains to be seen if the enhanced performance of PTM also enhances activity of human T cells.

Besides CD28, the stimulatory domain of 4-1BB has also been explored as an addition to chimeric PD1 receptors. The first attempt to add a 4-1BB domain (AA214-255) to the PD1/28 receptor by Ankri *et al* failed due to minimal surface expression of the PD1/28/BB construct,^26^ but by inserting flexible G4S linkers between functional units, Tang *et al* generated a functional PD1:CD28:4-1BB receptor, which contains PD1 (AA1-167), CD28 (AA141-220) and 4-1BB (AA214-255).^29^ Without concurrent expression of a tumor-reactive CAR or TCR, the PD1:CD28:4-1BB receptor alone could efficiently direct human T cells to kill PD-L1-expressing tumor cells and control tumor growth in vivo in humanized mice.

Signaling domains from costimulatory receptors well studied in NK cells have also been used in PD1-based stimulatory chimeric receptor. Guo *et al* developed a chimeric receptor named PNBB composed of the PD-1 extracellular portion (AA21-170) fused to the hinge region, transmembrane and cytoplasmic domains (AA1–90) of NKG2D, and the 4-1BB signaling domain (AA214-255).^30^ Because NKG2D belongs to the type II family of transmembrane proteins, in which C-terminal domains locate extracellularly while N-terminal domains are intracellular, the extracellular portion of PD-1 was directly fused to NKG2D at the C-terminus after the NKG2D hinge region. Even though the PD-1 ectodomain orientation was reversed, the PNBB receptor still efficiently bound to PD-L1 and enhanced NK cell cytotoxicity against PD-L1-expressing human tumor cells in vitro. Unlike NK cells, in which NKG2D directly mediates killing of target cells, human CD8^+^ T cells constitutively express NKG2D, and use it as costimulatory receptor in the context of TCR activation to transduce activating signals through DAP10 and activate the PI3K-Akt pathway, suggesting a role in cell survival.^31^ Recognizing these differences in cell type, the mechanistic signaling and function of PNBB in human CD8^+^ T cells vs NK cells needs further clarification.

Other groups have combined both NK and T cell signaling domains to further augment T cell activation. Barber *et al* developed a murine receptor called chPD1-Dap10 that contains the PD1 extracellular domain (AA1-155), the transmembrane region of CD28 (AA141-177), and the cytoplasmic domains of Dap10 (AA 57–79) and CD3ζ (AA52-164). They also constructed another receptor called chPD1-CD28, which replaced the Dap10 domain in chPD1-Dap10 with the CD28 signaling domain (AA178-218). Both receptors induced production of proinflammatory cytokines in T cells upon PD-L1 engagement. However, chPD1‐CD28 induced anti‐inflammatory cytokines like IL-10. Moreover, chPD1‐Dap10 induced a central memory T‐cell phenotype whereas chPD1‐CD28 induced an effector memory phenotype. chPD1‐Dap10-expressing T cells also showed enhanced in vivo persistence and antitumor activity compared with chPD1‐CD28-expressing T cells.^32^

Currently there are three ongoing phase I clinical trials using chimeric receptors based on PD1 and CD28 (NCT02930967, NCT02937844, and NCT03258047, table 1). Results of trial NCT03258047 have been published recently.^33 34^ In this trial, the PD1:CD28 receptor containing the AA141 cysteine has been tested in anti-CD19 CAR-engineered T cells for treatment of PD-L1-positive B cell lymphoma. Results show that CD19-CAR T cells expressing the PD1:CD28 coreceptor had superior T-cell proliferation, cytokine production, and killing of PD-L1^+^ B-cell lymphoma cells in vitro and in vivo compared with the prototype CD19-CAR T cells. The remaining two trials focused on PD-L1^+^ malignant tumors and glioblastoma multiforme are ongoing and no data has been released at this time.

**Table 1:** Recorded clinical trials of chimeric non-antigen receptors in ClinicalTrials.gov

Receptors	NCT#	Phase	Status	Condition or disease	Intervention/treatment	Sponsor	First posted	Refs
**PD1-CD28**	NCT02930967	1	Unknown	PD-L1 +malignant tumors	Autologous CSR T	China Meitan General Hospital, China	October 12, 2016	n/a
	NCT02937844	1	Unknown	Glioblastoma multiforme	Anti-PD-L1 CSR T cells Cyclophosphamide fludarabine	Beijing Sanbo Brain Hospital, China	October 19, 2016	n/a
	NCT03258047	2	Unknown	B cell lymphoma	CAR-T	First Affiliated Hospital of Zhejiang University, China	August 22, 2016	33 34
**CD200R-9aas-CD28cys**	NCT01640301	2	Active, not recruiting	Acute myeloid leukemia HLA-A*0201 Positive	WT1-sensitized allogeneic T-lymphocytes	Fred Hutchinson Cancer Research Center, USA	July 13, 2012	39
**NKG2D-based**	NCT04658004	1	Not yet recruiting	Acute myeloid leukemia	NKG2D CAR T-cells	Zhejiang University, China	December 08, 2020	n/a
	NCT03415100	1	Recruiting	Solid tumors	CAR-NK cells targeting NKG2D ligands	The Third Affiliated Hospital of Guangzhou Medical University, China	January 30, 2018	n/a
	NCT04550663	1	Not yet recruiting	Hepatocellular carcinoma Colorectal cancer Glioma	KD-025 CAR-T cells	The Affiliated Nanjing Drum Tower Hospital of Nanjing University Medical School, China	September 16, 2020	n/a
	NCT04717999	n/a	Not yet recruiting	Recurrent glioblastoma	NKG2D CAR-T	UWELL Biopharma, Taiwan	January 22, 2021	n/a
	NCT02203825	1	Completed	Acute myeloid leukemia myelodysplastic syndrome multiple myeloma	CM-CS1 T-cell infusion	Celyad Oncology SA, Belgium	July 30, 2014	66

n/a, not available.

#### CTLA4-based stimulatory chimeric receptors

CTLA-4 is an immune checkpoint targeted by several FDA-approved antibodies for cancer therapy. Shin *et al* reported a murine chimeric receptor called CTLA4-CD28 which contains the extracellular and transmembrane portion of CTLA-4 (AA1-189) and the cytoplasmic portion of CD28 (AA178-218). This CTLA4-CD28 receptor could compete with endogenous CTLA-4 to enhance T cell activation. The maximal anti-tumor effect was seen when CTLA4-CD28 was expressed in both CD4^+^ and CD8^+^ T cells.^35^ *Park et al* found the CTLA4-CD28 modification of T cells enhanced the therapeutic efficacy of donor lymphocyte infusion in a mouse model of acute lymphocytic leukemia.^36^ Perplexingly, a CTLA4-CD28 chimera has been shown to naturally exist in some T cell lymphomas. Yoo *et al* identified a fusion gene between CTLA4 and CD28 coding for the extracellular and transmembrane domains of CTLA-4 and the cytoplasmic region of CD28 and induced activation signals on T-cell stimulation. This observation raises a safety consideration that expression of an uncontrollable activation signal such as CTLA4-CD28 could transform adoptively transferred T cells.^37^

#### Other inhibitory-to-stimulatory chimeric receptors

The inhibitory receptors TIGIT and CD200R have been modified to generate stimulatory signals. TIGIT negatively regulates T and NK cell activation on binding to CD155 on tumor cells. Hoogi *et al* compared two designs of TIGIT/CD28 chimeric receptors called TIGIT-28-TM-TIGIT and TIGIT-28-TM-28, respectively. The former contained extracellular and transmembrane domains of TIGIT(AA1-165) fused to the intracellular tail of CD28(AA182-220); the latter used extracellular domains of TIGIT(AA1-134) fused to the transmembrane and intracellular tail of CD28 (AA140-220). TIGIT-28-TM-TIGIT was superior to TIGIT-28-TM-28 in surface expression and enhancement of CAR or TCR T cell function. The activation of such chimeric receptor signaling was dependent on CD155 expression on tumor cells, contributed to rescue of hypofunctional T cells in the TME, and mediated robust antitumor cytotoxicity in murine xenograft models when combined with tumor-reactive TCR.^38^ CD200 is expressed on various types of tumor cells and binds CD200R on T cells to inhibit T cell activation. Oda *et al* generated a panel of murine CD200R and CD28 fusion constructs expressed in the pMP71 retroviral vector, with variable alternations of transmembrane domain and length of stalk regions. They identified CD200R-9aas-CD28cys as the lead candidate in a T cell proliferation assay. This construct contains the extracellular portion of CD200R(AA1-229) fused to the AA142-218 region of CD28. The membrane proximal cysteine (AA142) of CD28 was incorporated in this optimal receptor to promote disulfide bond-mediated homodimerization and enhance native CD28 signaling. Modified T cells exhibited enhanced survival and activity of controlling tumor growth in a murine leukemia model. The human CD200R-9aas-CD28cys receptor also worked efficiently to augment CD8^+^ T cell proliferation and function on activation by CD200-expressing human tumor cells in phase 2 clinical trial (NCT01640301,table 1).^39^ Oda *et al* developed another receptor composed of the Fas ectodomain/transmembrane and 4-1BB intracellular tail, Fas_tm_-4-1BB, which can convert a death signal to a prosurvival signal to T cells and enhances T cell therapy for cancer.^40^

#### Inhibitory cytokine receptor-based stimulatory chimeric receptors

Several inhibitory cytokines and receptors are involved in preventing optimal antitumor T cell responses. The TME usually has high levels of IL-4, which contributes to immunosuppression by inducing polarization of Th2 cells and M2 macrophages while inhibiting Th1 cell polarization and proinflammatory cytokine production by antigen-presenting cells (APCs).^41^ In some tumor types, IL-4 directly promotes tumor growth.^42^ In order to overcome these inhibitory signals, Wilkie *et al* developed a chimeric IL-4 receptor called 4αβ, which contains the IL-4Rα ectodomain (AA1-233) fused to the transmembrane and intracellular domain of IL-2Rβ (AA241-551). Together with CARs, 4αβ was expressed within the SFG retroviral vector in human T cells. When introduced into the IL-2/IL-15-dependent CTLL-2 T cell line, 4αβ induced IL-2/15-like signaling in response to human IL-4. Primary human T cells expressing 4 αβ and various CAR constructs could be expanded exponentially in vitro and exhibited prolonged activation states and cytotoxic activity when cocultured with cognate CAR-antigen expressing target cell lines.^43^ Similarly, Mohammed *et al* generated a chimeric receptor called 4/7 inverted cytokine receptor (ICR) containing the IL-4Rα ectodomain (AA1-233) and the transmembrane and intracellular domain of IL-7Rα (AA240-459). When used alone, 4/7 ICR cells could respond to IL-4 and promote T cell proliferation but could not induce antitumor activity, but combing 4/7 ICR and CAR resulted in antigen and cytokine dependent antitumor T cell responses in vitro and in xenograft tumor models in humanized mice.^44^ Wang *et al* developed a similar receptor called 4/21 ICR which used the IL-4Rα ectodomain (AA1-233) and the transmembrane and intracellular domain of IL-21R(AA233-538), which was expressed via the lentiviral vector pRRLSIN. In the presence of IL-4, 4/21 ICR-expressing CAR T cells developed Th17-like phenotypes and exhibited enhanced persistence and antitumor activity.^45^

Transforming growth factor-β (TGF-β) is another immunosuppressive cytokine highly upregulated in the TME, inhibiting activation and function of effector T cells while inducing regulatory T cell differentiation. The TGF-β receptor is composed of a type II receptor dimer (TGF-βRII) and a type I receptor dimer (TGF-βRI). Dominant-negative TGF-βRII receptors have been developed to compete with wild-type receptors to enhance CAR T cell and NK cell effector function.^46 47^ Burga *et al* fused a DAP 12 kDa intracellular tail (AA62-113) to domain-negative TGF-βRII (AA1-199) to generate a chimeric receptor NKA. DAP12 is an ITAM-containing adapter protein that mediates activation signaling downstream of several receptors in both NK and myeloid cells.^48^ NKA expression in NK cells inhibited TGF-β signaling and induced activation signals in the presence of TGF-β resulting in enhanced activation phenotypes with superior antitumor efficacy in vivo in mice.^49^ NKA could also be used in T cell applications since DAP12 also contributes to T cell activation and function.^50^ Burga generated a synNotch-like receptor called NKCT that contains TGF-βRII ectodomains fused to the mouse Notch1 minimal regulatory region (AA1427-1752) coupled to the DNA-binding domain of RELA (NFκB p65) and a VP64 effector domain, which is supposed to be cleaved at membrane proximal sites and release the VP64-RELA transcription factor to nucleus on ligand binding. NKCT expression enhanced the NK cell activation after TGF-β exposure but was inferior to NKA in overall antitumor activity. Given thatthe synNotch receptor is believed to be activated by mechanical force induced by surface ligands or membrane-bound soluble ligands, it is not clear how exogenous soluble TGF-β activated the synNotch-like NKCT receptor in this case.

### Enhanced stimulatory receptors binding natural ligands

Various stimulatory receptors exist on T cells to augment their responses to endogenous signals. For therapeutic purposes, the intrinsic function of stimulatory receptors can be enhanced by genetically coupling distinct activation signals with known downstream profiles and strengths.

In a pioneer study for CD3ζ function in 1991, Irving and colleagues fused the extracellular portion and transmembrane domain of CD8α(AA1-208) to the intracellular tail of CD3ζ(AA52-163) to get a chimeric receptor called CD8/ζ. CD8/ζ could be expressed on T cell surfaces independent of TCR expression. Crosslinking CD8/ζ with antibodies was shown to activate T cells, a seminal finding in our understanding of TCR and CD3 function.^51^ Here CD8α was selected due to its ability to form homodimers, which could mimic CD3ζ’s dimeric state. Similarly, CD8/CD28 chimeras were generated by Stein et al in 1994 in a study for CD28 function. These chimeras contained the CD8α extracellular portion and transmembrane domain (AA1-208) fused to the CD28 intracellular domain (AA180-220). CD8/CD28 contributed to the activation of a CD28-deficient T cell leukemia line on antibody-mediated crosslinking.^52^ These early CD8-based chimeric stimulatory receptors have not been tested in tumor immunotherapy applications but provided essential knowledge of key stimulatory domains in T cells and lay the foundation for the development of CARs.

Given that CD8α is recruited to the supramolecular activation complex (SMAC) on TCR activation, we hypothesized that CD8α could be used as a carrier to couple MYD88 signaling with TCR signaling in an antigen-dependent manner. MYD88 is a potent pro-inflammatory mediator downstream of innate immune receptors including Toll-like receptors (TLRs) and the IL-1 family of cytokine receptors.^53^ CD8α:MYD88 fusion thus introduces innate immune receptor signaling to T cells upon TCR activation, providing extra fuel to T cell activation and function.^54^ Our lab generated CD8α:MYD88 chimeric receptors that contain the human or murine CD8α extracellular portion and transmembrane domain (AA1-203 from human CD8α; AA1-217 from murine CD8α) fused to the human MYD88 DD and intermediate domain (AA1-155). Importantly, CD8α:MyD88 costimulation functioned in a TCR-dependent but TLR ligand independent manner resulting in enhanced activity against weakly immunogenic or lowly expressed tumor antigens. CD8α:MyD88-expressing T cells improved antitumor responses in mice and was associated with TME alterations including generation of a unique tumor cytokine/chemokine signature, improved T-cell infiltration with reduced markers of T-cell exhaustion.

CD2 is a costimulatory and adhesion molecule expressed on T cells and NK cells that binds to CD58.^55^ It locates in the peripheral adhesion ring of SMAC during T cell activation. Feldhaus *et al* developed a human CD2/CD28 chimeric receptor composed of the CD2 extracellular portion (AA1-190) and CD28 transmembrane and intracellular domain (AA153-220). CD2/CD28 initiated CD28 signaling on CD58 binding in the absence of B7 ligands.^56^ Given the wide expression of CD58 on tumor cells, they hypothesized that CD2/CD28 could provide an efficient way to activate CD28 signaling in TME but follow-up studies with modern preclinical approaches have not assessed the utility of this construct.

CD4-containing chimeric non-antigen receptors have also been explored, particularly for treatment of HIV. Early CD4ζ constructs contained the CD4 ectodomain (AA1-396) fused to CD3ζ transmembrane and intracellular domain (AA31-164). HIV control was mediated through CD4-binding of viral proteins on infected cells, and did not affect uninfected cells even if expressing MHC-II.^57^ CD4ζ was shown to be modestly effective in phase II trials, but these results were overshadowed by these success of modern anti-retroviral therapies.^58^ Additional improvements were made by incorporating CD8α hinge and transmembrane domains (AA128-210), 4-1BB intracellular domains (AA214-255), CD3ζ intracellular domains (AA52-164), and the EF1α promoter for increased expression.^59^ So far these CD4-based chimeric receptors have not been tested in T cell-based cancer therapies.

A final stimulatory receptor under investigation is NKG2D. Natural ligands for NKG2D include the MIC and RAET1/ULBP families of proteins that are upregulated on stressed, infected, and transformed cells.^60^ Upon ligand binding, NKG2D on human NK cells and CD8^+^ T cells transmits activating signals through Dap10. Zhang *et al* fused CD3ζ intracellular signaling domains to NKG2D and Dap10 and tested the function of these chimeric receptors in murine T cells. They found these chimeric receptors were superior to natural NKG2D or Dap10 in their ability to respond to and kill NKG2D-ligand-bearing tumor cells.^61^ They also tested a human NKG2D:CD3ζ chimeric protein in human T cells and showed that CD8^+^ T cells engineered with NKG2D:CD3ζ could recognize and lyse NKG2D ligand-positive tumor cells more efficiently than control T cells with wild type NKG2D.^62 63^ NKG2D:CD3ζ also enhanced NK cell activation and killing of tumor cells.^64^ However, VanSeggelen *et al* found NKG2D fused to CD28 or CD3ζ could make T cells generate lethal toxicity in mice on adoptive transfer, raising concerns about the off-tumor toxicity of the clinical application of NKG2D-based chimeric receptors.^65^ Great care must be taken when artificially increasing activating signaling in T cells. Five clinical trials based on NKG2D chimeric receptors have been reported (NCT04658004, NCT03415100, NCT04550663, NCT04717999, NCT02203825, table 1). In the completed phase I trial NCT02203825, human NKG2D:CD3ζ chimeric receptor was tested in patients with acute myeloid leukemia, myelodysplastic syndrome, or multiple myeloma. Results showed that a single infusion of NKG2D:CD3ζ T cells without prior lymphodepleting therapy was safe, with no dose-limiting toxicities, cytokine release syndrome, or CAR T cell-related neurotoxicity observed. In one patient with AML that received the highest dose, hematological parameters were transiently improved, and disease stability without subsequent treatment was observed in several other patients, suggesting some positive treatment effects. However, expansion and persistence of NKG2D:CD3ζ T cells in vivo was limited.^66^

### Synthetic receptor-ligand pairs

The activity of chimeric receptors binding to natural ligands is restricted by the physiologic source of ligands and competition from endogenous receptors for ligands. Furthermore, such chimeric receptors cannot be selectively activated by exogenous natural ligands. Orthogonal synthetic receptor-ligand pairs, in which both the receptor and ligand are distinct from their natural counterparts, could be a solution to these issues. There have been several attempts to engineer fully synthetic receptor-ligand biologic systems.

Erika *et al* developed synthetic cytokine receptors (SyCyRs) composed of high affinity green fluorescent protein (GFP)-nanobodies and monomeric red fluorescent protein (mCherry)-nanobodies fused to transmembrane and intracellular domains of GP130 and IL-12Rβ1/IL-23R, respectively. Homodimeric and heterodimeric GFP:mCherry fusion proteins were used as synthetic cytokine-like ligands. These ligands induced dimerization of IL-12Rβ1/IL-23R heterodimers, IL-23R homodimers, or GP130 homodimers, and subsequently activated JAKs and STAT3.^67^ IL-23- or IL-6- like signaling could be induced by SyCyRs in tumor cell lines. Although SyCyR-induced IL-23-like signaling might not be as efficient as IL-23, IL-23 engineering can improve CAR T cell function in solid tumors,^68^ While still in early development, various types of SyCyRs could be generated to enhance T cell-based cancer therapy. Unfortunately, there is evidence to suggest GFP and other haptens are immunogenic and thus tolerability of fully synthetic approaches is questionable.^69^

Garcia *et al* engineered an orthogonal mouse IL-2 cytokine-receptor mutant pair consisting of an orthoIL-2/IL-2Rβ pair. In this construct, orthoIL-2 contained several substitutions including E29D, Q30N, M33V, D34L, Q36K, E37A, while orthoIL-2Rβ contained H134D and Y135F mutations. These substitutions create a faithful association in which the mutated ligand and receptor only bind to each other and not to native proteins. They do not interact with the natural IL-2 receptor even in the presence of CD25. Such high specificity reduces off-target effects and toxicity that is usually induced by high-dose natural IL-2 treatment. Further, unlike natural IL-2, orthoIL-2 does not contribute to generation and function of endogenous Foxp3^+^ regulatory T cells. In mice with intact immune systems, orthoIL-2 supports the selective expansion of orthoIL-2β-engineered T cells but not wild-type T cells and augmented cytotoxic T cell function in an orthoIL-2Rβ-dependent manner. In the B16-F10 mouse model of melanoma, orthoIL-2Rβ-engineered pmel-1 T cells with orthoIL-2 treatment had comparable effects with natural IL-2-treated wildtype pmel-1 T cells regarding control of tumor growth. However, orthoIL-2 had no therapeutic benefit in mice that received wildtype pmel-1 T cells, indicating that orthoIL-2 activity is restricted to orthoIL-2Rβ-engineered pmel-1 T cells.^70^

In T cells, it has been reported that ectopic expression of GPCRs like CXCR1, CXCR2, CXCR6, or CCR4 can enhance migration and persistence of CAR T cells in tumor and augment therapeutic efficacy of CAR T cells.^71–73^ Functional pairs of mutated GPCRs and nonendogenous ligands have great potential as synthetic tools to engineer T cells for cancer therapy. These engineered GPCRs and ligands are also called receptors activated solely by a synthetic ligand (RASSLs).^74 75^ One application of RASSLs in T cells has been reported by Lim *et al*, in which an orthogonal pair of a Gαi-coupled receptor Di and a bioinert drug-like small molecule, clozapine-N-oxide (CNO), was able to direct human T cell migration to the sites with CNO slow-release microspheres in mice.^76^ CNO is an inert metabolite of the FDA-approved antipsychotic drug clozapine, and lacks pharmacological activity in vivo at non-DREADD targets.^77 78^ Using the CNO-based RASSL system could be a promising approach to redirect engineered T cells to tumor sites. This system also served as a potential bioswitch to control T cell signaling.

## Conclusions

Owing to developments in T cell biology, bioengineering, and biotechnology, T cell engineering for cancer therapy is rapidly evolving. The modular design of natural immune receptors and their signaling domains allows them to be isolated and transplanted into synthetic molecules with specific functions in mind. Chimeric non-antigen receptors have been developed together with tumor antigen-receptor CARs and TCRs and have great potential for improving therapeutic efficacy of T cell-based cancer therapy. When designing a receptor for T cell-based cancer therapy, one should be aware that synthetic domains could lose regulation from networks that govern their natural counterparts. Additionally, unexpected regulation could occur when different domains are arbitrarily fused together as a chimeric protein. All properties of such unnatural biological elements including function, regulation, and immunogenicity should be carefully evaluated before human application.
